# When Children Become Adults: Should Biobanks Re-Contact?

**DOI:** 10.1371/journal.pmed.1001959

**Published:** 2016-02-16

**Authors:** Noor A. A. Giesbertz, Annelien L. Bredenoord, Johannes J. M. van Delden

**Affiliations:** Department of Medical Humanities Julius Center, University Medical Center Utrecht, Utrecht, The Netherlands

## Abstract

Noor Giesbertz and colleagues consider different re-contact policy designs that could be used by biobanks to obtain permission for the continued use of samples collected from children.

**Summary Points**

- Children’s samples are usually included with parental permission, and there is no clear guidance on whether participants should be re-contacted at maturity to obtain their permission for the continued use of their samples.
- Respect for autonomy and protection of privacy are important arguments in favor of re-contacting participants at maturity.
- There are four re-contact policy designs that could be considered, ranging from a thin opt-out policy (participants can withdraw their samples, but the biobank does not re-contact the participant) to a strict opt-in (samples will be destroyed when participants do not give their consent).
- We suggest that biobanks adopt a thick opt-out as the default re-contact policy, which means that biobanks re-contact children at maturity and give them the opportunity to withdraw their samples.

## Introduction

At the moment, there are many collections of human biological samples stored for medical—scientific research purposes that include samples from children [1]. These pediatric biobanks facilitate research, which is considered important for improving (pediatric) health care by generating biomedical knowledge [2,3]. However, pediatric biobank research gives rise to specific ethical issues. At the time of inclusion, many children cannot, or are legally not allowed to, consent for themselves, and typically parental permission is required. Samples may still be stored and used by biobanks when children become autonomous adults. The question arises whether children should be re-contacted to obtain their own consent, or give the opportunity to withdraw their samples, when they reach adulthood. Often, this is referred to as re-consent [4–6]. This term, however, is a misnomer, since the child has not consented in the first place. We therefore use the terms re-contact and consent.

In practice, biobanks have adopted different approaches to re-contact and consent. A study on six birth cohort studies found that only the cohorts that follow children into adolescence or past childhood recognize a need to seek consent as the child matures [7], or biobanks attribute a role to parents/guardians to inform their child about the tissue that has been stored and used for research [8]. Furthermore, our international case study on consent procedures in pediatric biobanking [9] shows that regulation plays a key role when pediatric biobanks design their consent procedures. However, major guidelines do not provide sufficient guidance on re-contact and consent [10–12], and there is only very limited literature that analyses the issue in depth [4–6,13].

Given the fact that biobanks already include pediatric samples, and in light of the rapid developments in biobank research, it is important to address the issue of re-contact and consent now. In this paper, we discuss the arguments in favor and against re-contacting participants at maturity and examine different re-contact policies that can be considered.

## Arguments in Favor of Re-contact

A key argument in favor of re-contacting a participant who donated tissue as a child is respect for autonomy. Autonomy can be interpreted in a negative and positive account. In a negative account, autonomy refers to an individual’s right to make one’s own decisions without undue influence or coercion from others [14–16]. Positive autonomy entails the ability and ideal to take control over one’s life and to live according to one’s values and beliefs [14–16]. In a research setting, negative autonomy means that a person has the right to participate in a study only with her authorization, and the promotion of positive autonomy enables a potential research participant to decide whether participating is in accordance with her own values and believes. Informed consent is obtained to respect and promote autonomy and to protect the participant from harm [17].

A challenge in pediatric biobanking is that immature children are generally not capable of deciding on research participation, and informed consent cannot be obtained. When children are included in scientific studies, other safeguards are put into place to protect them such as parental permission. Although parents cannot exercise their child’s autonomy, they can protect the child’s future autonomy rights by respecting the child’s right to an open future. Taken as a negative anticipatory autonomy right, this principle requires that no unnecessary irrevocable decisions are made for the child [18]. The right to an open future principle indicates that a child should have the prospect of being contacted in the future to decide for herself on continued participation. In addition, biobank research may yield results that can be of interest for participants on a personal level. Particularly, the return of individual genetic research results in the context of next generation sequencing (NGS) of DNA has been discussed extensively in the literature [15,19]. Grounded in a child’s right to an open future, a child should decide if and which type of genetic information is given once she reaches maturity [20,21], and re-contact will enable the participant to decide on the return of individual genetic information. Once the participant has acquired the capacity to make an autonomous decision on research participation, her—then developed—autonomy can be respected and promoted by contacting her and obtaining informed consent.

One could argue that autonomy should not be given so much weight in biobank research. After all, tissue research differs from research involving participants themselves, especially when samples are anonymized. However, the vast majority of biobanks will store samples coded, since they will be most valuable for research when linked to information about the person [22]. Also, it can be questioned whether complete anonymization of biological material and datasets would be possible at all [23]. Since there is still an identifiable link between participants and their material, and study projects may infringe on a participant’s personal values, it is important that they have a say in its use.

In line with respect for autonomy is the right to privacy. In biobank research particularly, informational or data privacy—the ability of the participant to exercise control over information about herself—is at stake [24]. Obviously, a preliminary requirement for exercising control and ensuring that personal data flow appropriately is awareness that one’s samples and data are included in a biobank. By re-contacting and informing the participants, they will be given awareness and opportunity to decide whether they want to be part of this biobank [5,6,25].

Re-contacting may be beneficial for biobanks as well. In case one considers the scope of parental permission as limited, re-contacting may be beneficial for biobanks by enhancing research possibilities. It has been argued that there should be certain restrictions on the use of pediatric samples when they are included with parental permission only: publicizing full genomes [4] or sharing samples and/or data by population biobanks [26] should only take place based on a participant’s personal consent. Although, at the moment, there is no consensus on the limits on the use of pediatric samples [26–30], when restrictions are accepted, re-contacting the child will provide the opportunity for biobanks to expand their research possibilities.

Another advantage of re-contact is that it provides the opportunity to actively involve and engage the participant in research. It acknowledges that translational research cannot be conducted without the involvement of individuals willing to act as research participant. The longitudinal character of biobanking and the unknown future studies at the time of sample inclusion challenge traditional models in research ethics. Instead of a one-time agreement at the start of a research project, continuous approaches such as an authorization model with an opt-out clause are increasingly proposed. Such a model provides individuals with the opportunity to check whether what they consented to is actually happening and vice versa [31]. This model can promote more informed, active, and critical participants by creating the conditions for citizens to reflect on their participation in biobanks [31]. This argument is linked to a broader ideal of scientific citizenship, understood as the ideal of active citizens who are well informed and enabled to make decisions about scientific research [15,16,31]. Scientific citizenship may lead to better protection and promotion of the research participants’ interests [16,31]. It is linked to the deliberative democratic ideal that citizens are not passively subjected to policies but rather are provided with the opportunity to act and coshape, though they are not necessarily required to do so [31].

## Arguments against Re-contact

Re-contacting participants, however, can hamper research in several ways. First, it will take time and requires a logistic and financial investment, especially when there is no follow-up contact planned for the study itself [5]. Re-contact may not even be feasible, for example when a participant has moved or passed away. Second, research can be hampered when participants refuse the continued research on their data and tissue. This may introduce bias and impair research quality since the number of available samples decreases. However, a decrease of samples as an effect of the refusal for continued participation does not seem a strong reason against re-contact. After all, this is the reason why re-contact is implemented in the first place: so people can decide whether they want to continue participation. Moreover, two studies that used hypothetical scenarios to investigate the willingness of adults to provide consent for the continued use of their pediatric samples concluded that a majority of their respondents would support continued research [22,32].

It is, however, undeniable that re-contacting participants will cost biobanks certain efforts, and in some cases will be difficult or impossible. The hampering of biomedical research does not only affect the interests of biobanks and researchers. Biobanks aim to facilitate research on biological samples, which can generate knowledge that may be used to improve health care. The development of health care is of interest to us all. Hence, when a re-contact policy hampers biomedical research, this may have serious societal costs and at the same time violate individual interests in, for example, the development of vaccines or new drugs.

Another argument against re-contact is that the participant may find it intrusive to be re-contacted for something that happened many years ago. Particularly, when material was obtained during an intense period of her life, for example when she had cancer in childhood, being re-contacted about this period may cause emotional distress. This could, however, be the other way around as well: cancer survivors may be very eager to contribute to research. In addition, many biobanks, for example population biobanks, will include material from healthy persons for whom the burden may be minimal. In addition, informing parents and participants about the re-contact policy might ease the burden.

## Discussion: Re-contact Policy and Practice

Strong arguments exist to support the view that participants should be re-contacted at maturity and give them the opportunity to decide on continued biobank research on their material themselves. However, at the same time, important arguments against re-contact should be taken into account when designing a re-contact policy. When shaping a re-contact policy, both the design of the procedure and the time of re-contact need to be considered.

### Design of Re-contact Procedure

At least four designs can be considered: ranging from a thin opt-out policy to a strict opt-in (Table 1). At one end of the spectrum is a thin opt-out (policy I): children can withdraw their samples and/or data, but re-contact is not initiated by the biobank. This policy would require little effort from biobanks, but the participant’s autonomy is hardly respected, and protection of a participant’s privacy is difficult. After all, how can someone exercise the right to withdraw when she is not aware of her inclusion in a biobank? It could be reasoned that parents, instead of the biobank, need to inform their child about the biobank and the possibility to withdraw. Although involving the parents should certainly be encouraged, we think that the responsibility to inform the participant cannot be transferred to the parents (completely). The biobank uses samples of an individual who is now capable to decide for herself and therefore has responsibilities towards that person. Although the thin opt-out procedure would be beneficial for biobank research (and consequently the possible development of health care), it hardly respects the participant’s autonomy. Therefore, we recommend that it should not be adopted in general. Instead, we advocate that only in exceptional situations, for example, where the expected value of the research is very high and re-contact is not feasible, a thin opt-out procedure is justifiable. An independent party such as a Research Ethics Committee (REC) should judge whether such an exception is appropriate.

**Table 1:** Re-contact policies for biobanks.

**Policy I**	**Thin opt-out**: re-contact is not initiated by the biobank, but children can withdraw their samples and/or data.
**Policy II**	**Thick opt-out**: children are re-contacted once they reach maturity and given the opportunity to withdraw. If children do not withdraw, the samples and/or data may still be used in accordance with the earlier obtained parental permission.
**Policy III**	**Best effort opt-in**: children are re-contacted and asked for consent. If children cannot be located or do not answer, the samples and data may still be used in accordance with the earlier obtained parental permission.
**Policy IV**	**Strict opt-in**: children are re-contacted and asked for consent. If children cannot be located or do not answer, the samples and data will be destroyed.

On the other end of the spectrum is a strict opt-in (policy IV): children are re-contacted and asked for consent; if they cannot be located or do not answer, the samples and data will be destroyed. It is to be expected that this policy hampers biobank research considerably. Not only will the material from individuals who are against participation be destroyed, the samples from persons who are indifferent to participation and do not want to put an effort into consenting will be excluded as well. In the end, a balance needs to be struck between enabling research and protecting and respecting individuals. Such a strict opt-in procedure seems disproportionate in many cases of biobank research, at least for the types of tissue research that are unlikely to infringe on personal values of the participant and do not entail high risks [33]. However, it should not be overlooked that there may still be certain risks involved in biobank research such as psychological and social risks linked to information that can be generated, stored, and used by biobanks [16,34]. These informational risks will be higher depending on the type of biobank, for instance, biobanks that generate ample identifiable genetic data or study a small population with specific characteristics. Although the fourth policy seems too strict for most biobank research, this policy may be appropriate when the risks are deemed higher. Also, when biobank practices are more likely to infringe on personal values, for example the creation of chimeras or when commercial interests are involved, policy four may be more appropriate. Further interdisciplinary debate and studies on people’s views about different types of biobank research would be valuable to determine when a strict opt-in policy is required.

For biobank studies that do not require a strict opt-in, a thick opt-out (policy II) or a best effort opt-in (policy III) appears to be most suitable. A thin opt-out procedure (policy I) encompasses a very basic, plain procedure where biobanks or researchers hardly invest in enabling participants to actually opt-out if they want. Contrary, a thick opt-out (policy II) encompasses much more substantial efforts from biobanks or researchers to enable participants to decide to opt-out. Whereas a thin opt-out procedure merely offers participants the possibility to opt-out, a thick opt-out procedure requires that the following conditions are fulfilled: (1) awareness about someone’s involvement in a study has to be raised, (2) sufficient information about the study has to be provided, and (3) a genuine possibility to object has to be offered, and this objection has to be administered in such a way that it will be implemented [16]. With a best effort opt-in (policy III), children are re-contacted and asked for consent. If children cannot be located or do not answer, the samples and data may still be used in accordance with the earlier obtained parental permission. Although in policy II and III the autonomy and privacy of those people who could not be located would not be respected, we consider this a more appropriate balance of research versus individual interests than is the case with a thin opt-out procedure (policy I).

Thus, in both policy II and III, the use of samples from people who cannot be reached will be continued, and in either policy it is necessary to discuss which efforts biobanks should put into re-contacting a participant. The difference, then, between a thick opt-out and a best effort opt-in is very small. Both approaches require effort from the researchers to contact the participant. The key distinction is that while in policy II the participant is asked to refrain from acting to continue participation, in policy III she is asked to explicitly consent. An argument to prefer an opt-in procedure over an opt-out is that this generates evidence that someone agrees to participate in a study [16], and the main concern of an opt-out procedure is the possibility of using samples from people without their knowledge and possibly against their wishes [16]. However, this concern can only be addressed with a strict opt-in (policy IV) and not with a best effort opt-in (policy III): with a best effort opt-in, samples from people who could not be reached are also used. Moreover, it might even be confusing with the third policy that both samples from people who opt-in, as well as samples from people who refrain from acting (those who could not be reached), are used. Hence, a thick opt-out seems a more consistent and clearer policy. Therefore, we suggest that biobanks adopt a thick opt-out as the default re-contact policy, which means that biobank re-contact children at maturity and give the opportunity to withdraw their samples (policy II).

### Timing of Re-contact Procedure

The timing of re-contact needs attention as well. The capacity to make an autonomous decision is expected to develop gradually during childhood. We have previously argued in favor of a personalized approach when we discussed assent procedures in pediatric research. This means that both the assent procedure and the timing are adjusted to the individual development of a child, which varies because some children are more mature than others due to personal circumstances, such as hospital experience [35]. Such an individualized approach for re-contact is, however, unworkable when there is no ongoing contact between a biobank and its participants. Hence, setting an age limit seems a more feasible route. Obvious age limits are 16, 18, or 21 years, because these are established by precedent as signifying legal transitions into adulthood, e.g., obtaining a driver’s license or choosing who to marry. Different jurisdictions and national contexts may influence the age limit, but also biobank characteristics may influence when participants are expected to be sufficiently competent to make a decision on the continued use of their samples: immunological research on a blood sample, for example, is probably easier to understand than extensive genetic research and a qualified disclosure policy for individual research results.

## Challenges Ahead

We make an appeal to the biobank community to be aware that moral responsibilities do not end when assent and parental consent are obtained at inclusion of children’s samples. In recognizing the longitudinal character of their research, we suggest that biobanks design a re-contact policy in order to obtain participants’ permission at maturity for the continued use of their samples. We suggest a thick opt-out procedure (policy II) should be the default: when children’s samples are included in a biobank, the participants are re-contacted at maturity and given the opportunity to withdraw their samples. This implies that infrastructures need to be developed to support a thick opt-out procedure, for example web portals to communicate with participants. Furthermore, interdisciplinary debate, in which both biobank participants and researchers are represented, would be valuable to determine what best effort actually means for specific biobanks. It is a demandingness question that needs to be answered: how many efforts of certain biobanks are reasonable? In addition, further ethical reflection and empirical studies are needed to determine when it is appropriate, or necessary, to deviate from the default procedure. At the moment, pediatric biobanks should at least introduce one of the discussed re-contact policies and inform parents and children at the time of sample inclusion about this policy.
